# Altered motion repulsion in Alzheimer’s disease

**DOI:** 10.1038/srep40946

**Published:** 2017-01-20

**Authors:** Yan Li, Shougang Guo, Yongxiang Wang, Huan Chen

**Affiliations:** 1 Department of Neurology, Luoyang Central Hospital Affiliated to Zhengzhou University, Luoyang City, 471000, China; 2 Department of Neurology, Shandong Provincial Hospital Affiliated to Shandong University, Jinan City, 250021, China; 3 Department of Neurology, The First People’s Hospital of Jinan City, Shandong Province, 250011, China

## Abstract

Recent research in Alzheimer’s disease (AD) indicates that perceptual impairments may occur before the onset of cognitive declines, and can thus serve as an early noninvasive indicator for AD. In this study, we focused on visual motion processing and explored whether AD induces changes in the properties of direction repulsion between two competing motions. We used random dot kinematograms (RDKs) and measured the magnitudes of direction repulsion between two overlapping RDKs moving different directions in three groups of participants: an AD group, an age-matched old control group, and a young control group. We showed that motion direction repulsion was significantly weaker in AD patients as comparing to both healthy controls. More importantly, we found that the magnitude of motion repulsion was predictive of the assessment of clinical severity in the AD group. Our results implicate that AD pathology is associated with altered neural functions in visual cortical areas and that motion repulsion deficit is a behavioral biomarker for the tracking of AD development.

Alzheimer’s disease (AD) is typically characterized as progressive loss of cognitive functions such as worsened short-term memory and spatial cognition^1^,^2^. However, recent evidence indicates that sensory and motor changes might precede the cognitive impairments^3^,^4^, and hence they may provide an early behavioral biomarker for the risk of developing AD (for a recent review, see Albers *et al*.^5^). Along this line, deficits of visual perceptual functions, in particular visual motion processing, have been widely identified in AD patients^6^,^7^,^8^,^9^,^10^. For example, it is shown that AD patients had impaired motion sensitivity and speed perception as comparing to healthy elderly subjects^11^,^12^. AD patients also exhibit reduced ability to infer the direction of self-motion from the pattern of retinal optic flow in spatial navigation tasks^13^,^14^.

In parallel to motion processing deficits at the behavioral level, motion evoked potentials recorded from visual cortex are found to be weaker or even absent in AD patients^15^. Comparing to healthy controls, AD patients show reduced activations in area V5 and parietal-occipital cortex in a previous functional magnetic resonance imaging study^16^. Recently, there is an emerging view that many neurological disorders including AD might be critically associated with altered cortical connectivity^17^,^18^,^19^ and disrupted neural inhibition^2^,^20^,^21^. However, the functional relevance of these impaired inhibitory processing on the perceptual dysfunctions in AD remains largely elusive.

One compelling phenomenon demonstrating inhibitory functions in visual motion processing is the so-called direction repulsion^22^,^23^,^24^,^25^. It is characterized by illusory, enlarged angle perception between two stimuli moving in similar directions. This bias in direction perception can even take place when there is only one single motion direction^26^. The magnitude of repulsion measured in the single motion condition was reported to be smaller (about half) and it is dubbed as reference repulsion. Comparing to the two-stimulus repulsion, the phenomenon of reference repulsion is more variable and less established, and it is still under debate whether reference repulsion really exists^27^. It can be viewed that both types of repulsion may arise from the inhibitory interactions between neighboring neurons tuned for similar directions in motion sensitive cortical areas^22^,^28^,^29^, with the two competing directions are either both external (two-stimulus repulsion), or one external and one internal cardinal direction (reference repulsion).

In this study, we want to test the hypothesis that inhibition-based motion repulsion is altered in AD patients and that motion repulsion deficits are predictive of AD severity. We employed the two-stimulus repulsion because reference repulsion is more vulnerable to measurement noise and is less optimal for the current study with clinical participants. We will compare the magnitudes of motion repulsion in three groups of participants comprising of AD patients, age-matched healthy elderly, and young healthy adults. In addition, we will use inter-subject variability to examine the correlation between the magnitudes of motion repulsion and pathology severity in the AD group.

## Material and Methods

### Subjects

This study involved three groups of participants, each group with 20 participants. The demographic of these participants was summarized in Table 1. For the AD group, Each patient was selected based on the National Institute of Neurological and Communicative Disorders and Stroke–Alzheimer’s Disease and Related Disorders Association criteria (NINCDS-ADRDA) for the diagnosis of AD^30^. Prior to the experiment, we assessed the dementia levels for each subject based on the Mini Mental State Exam (MMSE score)^31^. The AD patients’ MMSE scores were no greater than 26. All non-AD participants had a MMSE score greater than 27 (the maximum MMSE score is 30). We recruited another two groups of healthy participants as the control populations; one consists of age-matched old control (OC), most of them are the spouse or relatives of AD patient, and the other group made up of young adult control (YC). All subjects had normal or corrected-to-normal vision and were free of other age-related common eye disease such as cataract and glaucoma based on ophthalmologic examination. All subjects (or their relatives) had provided an informed consent for their participation in the study before the experiment, which was conducted in accordance with and approved by the local Ethics Committee in the Provincial Hospital Affiliated to Shandong University.

### Visual stimuli and direction estimation task

**Figure 1:**
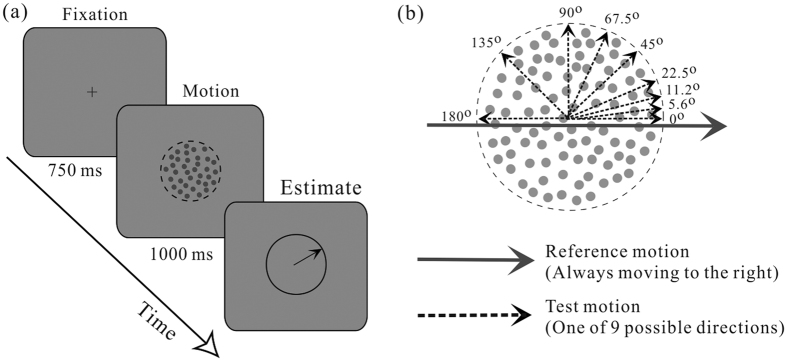
Direction estimation task. (**a**) Overlapped RDKs with different moving directions were presented foveally and participants are asked to estimate the perceived test direction after RDK offset by aligning the line with a mouse. (**b**) A range of test directions used in the experiment.

The visual motion stimuli were random dot kinematograms (RDKs) used widely in previous studies^32^,^33^,^34^. The RDKs were made up of a cloud of black dots (each 0.1 deg in diameter, 3.5 cd/m^2^) moving within an invisible circular area on a uniform gray background (23.6 cd/m^2^). The dot density was 5 dot/deg^2^ and movement speed was 3 deg/s. The RDKs were 5 deg in diameter. Each moving dot had a life time of 200 ms. There were two sets of dots within the RDKs (see Fig. 1b). The first set of dots was always moving to the right (0°) and served as the reference direction. The second set of dots moved at different directions from trial to trial and participants had to estimate its direction (test direction) after RDK offset. There were 9 possible test directions: 0°, 5.6°, 11.2°, 22.5°, 45°, 67.5°, 90°, 135°, 180°.

The RDKs were displayed on a CRT monitor (screen resolution: 800 × 600; refresh rate: 100 Hz). The CRT was 57 cm away from the observer whose head was stabilized by a chin rest. We used Matlab in together with the Psychophysical Toolbox^35^,^36^ to generate the RDKs stimuli and implement the control of experimental flow. Before data collection, each participant was given a few minutes to practice and familiarize with the task. As schematized in Fig. 1a, each trial was initiated after an ocular fixation of 750 ms at the central cross, followed by a foveal presentation of RDKs for 1000 ms. After RDKs offset, a circular ring with a line extending from the central fixation point were shown on the screen. Participants were required to report their perceived direction of the test motion (2^nd^ set of moving dots) as accurately as possible by rotating the line using a mouse. Once they have completed the estimation, they confirmed it by a button press which terminated the trial. No feedback was provided in each trial. Constrained by the limited length of the experiment with clinical patients, we followed methods of the previous study^37^ and performed a single measure per test-reference direction from each participant.

### Data analysis and statistics

We performed the data analysis using custom-made MatLab scripts. The magnitude of direction repulsion at each test direction was determined as the angular difference between the perceived and physical directions. We conducted mixed-design two-way ANOVA on the magnitudes of repulsion, with the test direction as the within-subject factor and the group as the between-subject factor. Note that the young participant group was intended to replicate the phenomenon of motion repulsion, allowing us to compare it with previous studies to validate our methods in general. The main group difference we want to explore is AD and OC groups. For this we performed unpaired *t*-tests on the repulsions at each test direction. The significance of all statistical analysis was defined at the level of p < 0.05.

## Results

### Reduced motion repulsion in AD patients

**Figure 2:**
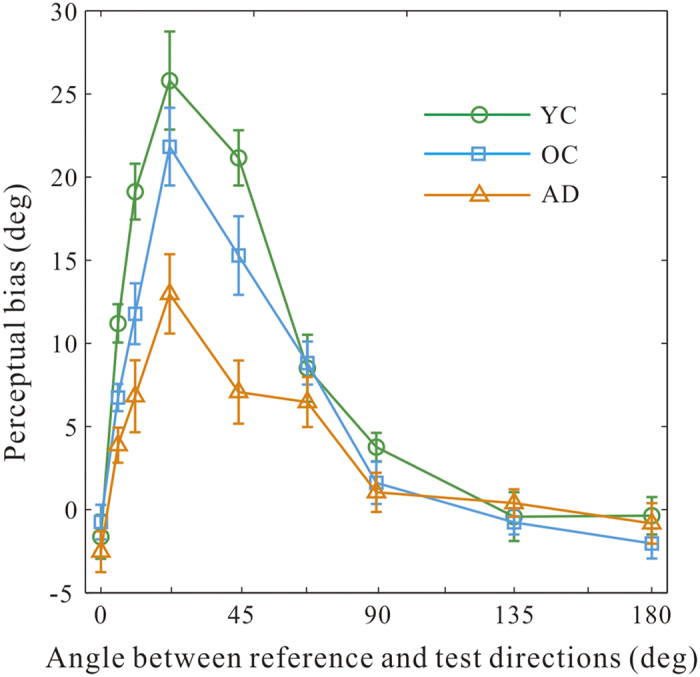
The dependence of the magnitudes of motion perceptual bias on the angular differences between the test and the reference direction in three groups. Results are across-participant mean ± SEM in each group.

We measured the magnitudes of motion repulsion as a function of angular separations between the reference and test directions in each group of participants (Fig. 2). There were several notable observations. Firstly, with the young healthy adults, we successfully replicated the phenomenon of motion repulsions which have been repeatedly shown in previous studies^22^,^23^,^24^,^25^. The perceived direction was misjudged, so that the reported angular difference between the reference and test motion was exaggerated relative to the actual angular separation (YC group, green line). The misperception was most pronounced for RDKs moving with angular differences at 11.2°, 22.5°, and 45°. Secondly, the patterns of motion repulsion in the AD and OC group were very similar to those in the YC group, except that their magnitudes exhibited a marked reduction. A mixed-design two-way ANOVA revealed that there were significant main effects on the direction (F(8,513) = 70.5, p < 0.001) and the group (F(2,513) = 29.3, p < 0.001), as well as a significant interaction between them (F(16,513) = 3.7, p < 0.001).

To find out if there are significant differences between AD patients and their age-matched elderly controls (OC), we performed a post-hoc unpaired *t*-test on the repulsion levels between OC and AD participants at each test direction. Our analysis showed that the differences reached significance levels at 5.6°, 22.5°, and 45° (ps < 0.05, df = 38). For the remaining directions, there was no significant difference between these two groups (ps > 0.05, df = 38).

### The magnitude of motion repulsion was correlated with AD severity

**Figure 3:**
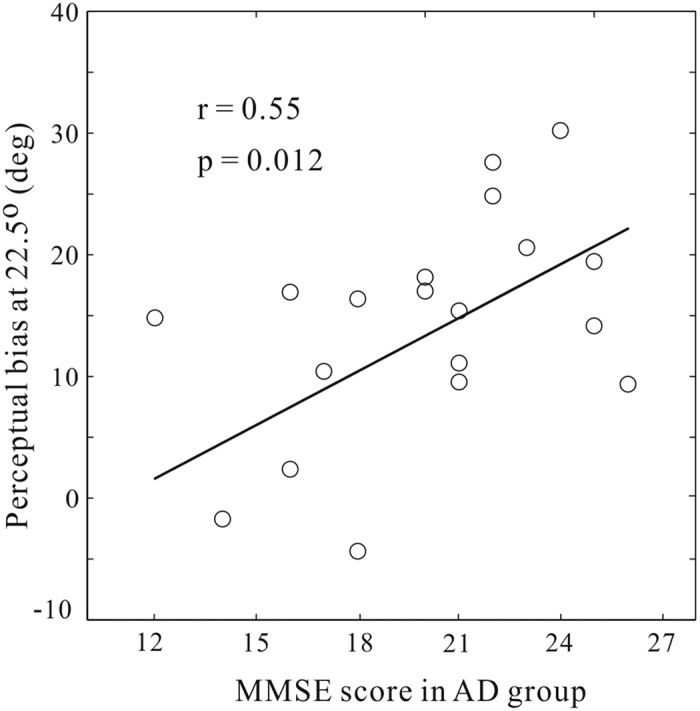
Correlation between the magnitudes of motion perceptual bias at 22.5° and the MMSE scores in the AD group. Each circle denotes data from one AD patient.

To gain insight into whether the magnitudes of motion repulsion were related to the presence of AD pathology, we exploited the inter-subject variability in AD patients and correlated the magnitude of their repulsions at 22.5° with the MMSE scores - a clinical assessment of AD severity (Fig. 3). Note that the MMSE scores from non-AD subjects exhibited little variability (see Table 1), and hence we conducted the correlation analysis only on the AD participants. We chose the angle 22.5^o^ because this was the direction we tested that elicited the largest misperception. Using the repulsion levels at neighboring directions produced qualitatively similar results. We found that the magnitudes of repulsion were positively correlated with the measured MMSE scores (Pearson’s linear correlation, r = 0.55 p = 0.012, df = 18). This means, those AD patients who scored less (more severe) had smaller repulsions in the perceived direction of the test motion. In other words, the performance in the motion estimation task can be used to predict and track the development of AD severity in the patient population.

## Discussions

In this paper we investigated the properties of direction repulsion in AD patients during visual motion perception. We found that the magnitudes of motion repulsion became significantly weakened comparing to healthy controls. Moreover, we showed that in AD patients motion repulsion levels were positively correlated with the clinical severity of the AD symptom. These results imply that AD is associated with impaired inhibitory processing in motion-sensitive visual cortical areas.

### Visual deficit versus general cognitive decline?

Since AD is known to cause multiple cognitive deficits, it might be concerned that the observed differences on the motion estimation task between AD patients and healthy controls are driven primarily by the cognitive deficits or other declined general mechanism, rather than a sensory deficit. For example, AD patients might have poorer understanding of the task or motor skills that is required to perform the task. However, we found this explanation is less likely. In our data, motion repulsion was smaller in AD patients, and this means that their estimations of direction were more accurate and less prone to the repulsion than the healthy controls. This counter-intuitively superior performance in AD patients cannot be explained by deteriorated cognition or motor skills. Another possibility of reduced motion repulsion in AD patients is that, during direction estimation patients might have adopted a biased response strategy that was different from the healthy controls. This interpretation is also implausible for two reasons. Firstly, the likelihood that all patients adopted the same strategy biasing towards “exaggerated” response is very slim. If there is a response bias, some patients might bias toward “exaggerated” response while others conservative response, then these response bias should average out on a population level. Secondly, we found that there exists a significant interaction between the test direction and the participant group in the magnitudes of motion repulsion (Fig. 2). The differences in the repulsion were present at some directions, but it disappeared when the actual angular separation was at 0° or larger than 90°. If differences in the response strategies play a role, then we should expect a global influence. But this clearly was not the case in our data. Taken together, we consider that the reduced motion repulsion in AD reflects a specific deficit in visual motion perception, not a general cognitive decline.

Why then do AD patients possess reduced motion repulsion? How does the current finding fit with the existing literature on visual motion perception? One speculation is that motion repulsion during direction judgments is special design of the visual system to optimize perceptual sensitivity. In our task, the test direction was perceived to be repulsed away from the horizontal line (the category boundary). As a result, it would be less likely to have across-boundary perceptual errors in the presence of sensory noise in the system^38^. To put it differently, the reduced motion repulsion in AD patients may lead to worsened perceptual sensitivity at the horizontal boundary. Whether this hypothesis is true or not remains to be tested. However, there was a recent perceptual learning study on motion repulsion in healthy subjects^39^, which imply that this assertion is highly plausible. In that study, the authors show that the magnitudes of motion repulsion are plastic and can be enhanced through visual perceptual training, and more intriguingly, the enhanced motion repulsion is associated with improved direction discrimination ability around the repulsion boundary. If we generalize these findings into the AD population, it might be that AD patients possess impaired motion repulsion (supported by findings from the current study), which then leads to reduced direction discrimination capacity. Further studies are needed to provide evidence for the latter speculation that links AD pathology with reduced discrimination ability around the repulsion boundary.

### Neural correlate of reduced motion repulsion in AD

Since motion repulsion is generally considered to reflect the inhibitory interactions between neighboring neurons that are tuned to similar motion directions^22^,^28^,^29^, it is conceivable that AD-related reduction in motion repulsion could be explained as the behavioral evidence implying cortical alterations of the inhibitory neural processing in motion-sensitive regions. Specifically, the lateral inhibition among neurons that are responsible for the phenomenon of motion repulsion might have weakened during the progression of AD development. This interpretation is in line with the notion that the aging^40^,^41^ and several other neurologic disorders^20^,^42^,^43^,^44^ are linked with disrupted inhibitory functions in the brain. The current findings extend this notion by suggesting that AD pathology also involves impaired inhibitory processing in visual motion cortical circuits.

However, it is yet to determine the inhibitory function at which specific motion-sensitive area(s) along the dorsal visual stream that give rise to motion repulsion. We know that motion information is first extracted in primary visual cortex (V1) at a local level and then passed on to the middle temporal area (MT) for integration and processing at a global level^29^. On the one hand, there is evidence showing that global motion perception drives direction repulsion^45^. This indicates that area MT is the site at which motion repulsion takes place, implying that AD pathology in the current study might be accompanied by disrupted inhibitory processing in MT. On the other hand, motion repulsion is reported to be monocular^37^ and is attenuated by binocular rivalry^46^. Since monocular neurons are present in V1^47^ but not MT^48^, this suggest that alterations of inhibitory functions in V1 are associated AD pathology. Future experiments should be designed to unambiguously decide and characterize the specific neural plasticity occurred in AD patients. In addition, as we outlined in the introduction, there are two types of motion repulsion and it is remained to be explored whether and how the properties of reference repulsion are altered by AD pathology. Reference repulsion and two-stimulus repulsion we used in the current study might have shared mechanisms. They may both arise from inhibitory interactions between directions; the difference lies in whether one of the directions is external or internal (e.g., imagery representation of the cardinal direction). A different view holds that reference repulsion is the result of the decoding strategy that optimizes fine perceptual judgments^49^. But either way we will need further studies to dissociate these two mechanisms and examine how they might go awry in AD patients.

## Additional Information

**How to cite this article**: Li, Y. *et al*. Altered motion repulsion in Alzheimer’s disease. *Sci. Rep.* **7**, 40946; doi: 10.1038/srep40946 (2017).

**Publisher's note:** Springer Nature remains neutral with regard to jurisdictional claims in published maps and institutional affiliations.

## Figures and Tables

**Table 1:** Demographics for the three groups of participants in this study.

	AD (n = 20)	OC (n = 20)	YC (n = 20)
Age (years)	68.2 ± 6.0	67.9 ± 7.3	25.9 ± 7.5
Female/male	11/9	10/10	10/10
MMSE score	19.8 ± 4.0	28.7 ± 0.8	29.0 ± 0.97

Age and MMSE scores are presented as mean ± std.
